# Evaluation of an Ionic Calcium Fiber Supplement and Its Impact on Bone Health Preservation in a Dietary Calcium Deficiency Mice Model

**DOI:** 10.3390/nu14030422

**Published:** 2022-01-18

**Authors:** Sara Elisa Herrera-Rodríguez, Eristeo García-Márquez, Eduardo Padilla-Camberos, Hugo Espinosa-Andrews

**Affiliations:** 1 Unidad Sureste, Centro de Investigación y Asistencia en Tecnología y Diseño del Estado de Jalisco, A.C., Km 5.5 Carretera, Sierra Papacal-Chuburná, Chuburná, Mérida 97302, Yucatán, Mexico; sherrera@ciatej.mx; 2 Unidad Noreste, Centro de Investigación y Asistencia en Tecnología y Diseño del Estado de Jalisco, A.C., Autopista Mty-Aeropuerto, Vía de la Innovación 404, Parque PIIT, Cd Apodaca 66628, Nuevo León, Mexico; 3 Biotecnología Médica y Farmacéutica, Centro de Investigación y Asistencia en Tecnología y Diseño del Estado de Jalisco, A.C., Av. Normalistas 800, Colinas de La Normal, Guadalajara 44270, Jalisco, Mexico; epadilla@ciatej.mx; 4 Tecnología Alimentaria, Centro de Investigación y Asistencia en Tecnología y Diseño del Estado de Jalisco, A.C., CIATEJ, Cam. Arenero 1227, El Bajío, Zapopan 45019, Jalisco, Mexico

**Keywords:** ionic calcium, fiber supplementation, osteoporosis, calcium-free diet

## Abstract

Ionic calcium can help in the prevention of the process of osseous decalcification. This study aimed to evaluate the physicochemical properties and toxic effects of ionic calcium-fiber supplement (*ICa^+^*) and its impact on bone health preservation in mice C57/BL6 fed a calcium-deficient diet. Physicochemical properties include FTIR, apparent calcium solubility estimated by the calcium ratio obtained by ionization chromatography and atomic absorption. In vitro genotoxicity and cytotoxicity of the *IC*a^+^ were assessed. Twenty-five 7-week-old C57/BL6 mice were fed calcium-free diet (CFD) or CFD plus CaCO_3_ (1.33 mg Ca) or CFD plus *ICa^+^* (1.33–6.66 mg Ca) for six weeks. After that, bone mass and microstructure parameters were assessed. Histological staining was performed to determine calcium deposits. *ICa^+^* (100%) exhibited an apparent calcium solubility higher than CaCO_3_ (12.3%). *ICa^+^* showed no cytotoxic and genotoxic in vitro activities. Histomorphometry analysis showed that the *ICa^+^* treated group displayed a higher trabecular number than the trabecular space. Also, the ratio BV/TV was increased compared with all treatments. Ionic calcium-fiber supplementation prevents bone deterioration compared to mice fed a calcium-deficient diet.

## 1. Introduction

Bone generation, remodeling, and turnover are continuous processes during the life of any vertebrate and were studied extensively for several decades. Bone remodeling depends on the activity of two specialized cells called osteoblasts and osteoclasts. The osteoblasts are responsible for secreting the organic part of the bone (osteoid matrix), which is mineralized, and the osteoclasts are responsible for bone matrix resorption [1]. The imbalance of bone remodeling can trigger some clinical diseases, such as osteoporosis or osteopenia [2]. The above generated significant interest in the mineral content due to its contribution as nutrients to the human diet. Particularly the contribution of free calcium or in the form of a soluble complex. Calcium is an essential element in living beings as a messenger of cellular signals in blood clotting, muscle contraction, nerve impulse transmission, and especially in the formation of the bone structure [3,4]. In general, the recommendations on daily calcium intake depend mainly on the age and sex of the person, varying from 200 to 1300 mg/day [2,4]. Calcium from food or dietary supplements is ionized in the stomach (Ca^2+^). Only then can Ca^2+^ be absorbed in the small intestine by transcellular transport and paracellular transport. However, the natural change of stomach pH (1.5–2.5) to the intestinal environment (5.5–6.5) can reduce the concentration of Ca^2+^, reducing the bioavailability of the various sources of calcium [5]. Reaching daily calcium requirements is often a difficult task to achieve, so the market for mineral supplements continues to grow day by day. Calcium salts and vitamin D are used to supplement dietary calcium deficiencies, including calcium carbonate, calcium phosphate, calcium citrate, calcium chloride, calcium glycerophosphate, calcium citrate tetrahydrate, and calcium gluconate [5,6,7].

When there is no proper calcium intake, the body usually obtains calcium from the bones to maintain their vital functions. Therefore, maintaining proper calcium homeostasis is vital to health [5,7]. However, consuming a low-calcium diet, people who consume high doses of antiacids, people with resections and shunts of the stomach, including bariatric surgery, people with atrophic gastritis, and older people may have several problems for calcium absorption [8]. Osteoporosis is a chronic degenerative disease characterized by low bone mass and deterioration of the microstructure of bone tissue [9,10], commonly classified as primary osteoporosis and secondary osteoporosis. Primary osteoporosis is the loss of bone mass during aging, while secondary osteoporosis is related to disease or medication intake [11,12]. Osteoporosis is one of the most recognized bone diseases worldwide for its high prevalence in women and men over 50 years old [9,10]. However, a low calcium intake in adolescents and young people is a matter of great concern, since their bone health at these stages of life could impact the development of bone disease in adulthood. Bone mineral density is a multifactorial problem such as race, sex, diet, eating habits, which can affect the health of bone tissue [13]. During the growth of children and adolescents, the bone mass index increases, so adequate calcium intake is required through food or calcium supplements [13,14]. Genetic, glucocorticoid treatments, and environmental aspects impact bone turnover in women, men, children, and adolescents [15,16]. Typically, pharmacological treatments against osteoporosis are based on calcium and vitamin D supplementation, antiresorptive agents (such as bisphosphonates, calcitonin, or denosumab), or anabolic drugs (teriparatide) [12,17]. Despite the differences between human metabolism and animal metabolism, animal models continue to be employed to understand the impact of new products that promote health in a short time.

Recently, nondigestible natural polysaccharides are regarded as the most promising prebiotics for bone health, e.g., galactooligosaccharides, oligofructose, fructans, resistant starch, and acacia gum [18,19,20]. Natural polysaccharides can resist enzymatic hydrolysis in gastrointestinal environments, being fermented in the distal sections of the large intestine by the microbiota (mainly lactobacillus and bifidobacterias). This process produces short-chain fatty acids, including acetate, propionate, and butyrate, which reduce the pH environment in the distal sections of the intestine, promoting calcium absorption [21,22]. For example, the fructans from *Agave tequilana Weber* var. Azul increased calcium deposition in the bones of male BALB/cAn Nhsd mice of five weeks old [23]. García–Vieyra et al. [24] observed that the supplementation with 10% of *A. tequilana* fructans increased calcium in plasma and reduced the trabecular bone loss of mice, increasing the bone density from 0.40 to 0.47 g/g. In a controlled study of calcium absorption in healthy adolescent men between 14 and 16 years, van den Heuvel et al. [25] reported that consuming 5 g of oligofructose three times a day positively affected calcium absorption. Calame et al. [26] observed that healthy human volunteers who consumed 10 g/day of gum arabic for four weeks significantly increased the content of bifidobacterias, bacteroides, and lactobacilli. Chitosan is a soluble dietary fiber that may increase gastrointestinal lumen viscosity. Chitosan might inhibit the uptake of dietary lipids by increasing the thickness of the boundary layer of the intestinal lumen. Lee et al. [27] reported that chitosan oligosaccharide had considerable bifidogenic potential, promoting bifidobacterias growth. These results showed the importance of nondigestible natural polysaccharides eating to preserve bone health. With this in mind, we kept a group of mice on a low-calcium diet to learn about the ability to employ a source of natural complex calcium-fiber supplement to preserve bone health. The calcium-fiber supplement complex is a natural blend of soluble fibers from *A. tequilana* fructans, chitosan, and gum arabic that keeps calcium ionized (*ICa^+^*) in intestinal environments. The purpose of this research is (1) to evaluate the physicochemical properties of *ICa^+^*, such as FTIR, elemental and Ca^2+^ content, as well as the hazardous potential of the *ICa^+^* by genotoxicity and cytotoxicity assays; (2) evaluate the impact of the *ICa^+^* into bone cancellous or trabecular bone, based on calcium absorption from an external source, provided by a calcium-free diet mice model C57/BL6. Calcium carbonate (CaCO_3_) was chosen as the reference control for in vivo test because it is a popular and accessible calcium supplement.

## 2. Materials and Methods

### 2.1. Materials

Ionic calcium-fiber supplement (*ICa^+^*) was produced according to Espinosa-Andrews and García-Márquez [28]. Briefly, the *ICa^+^* was produced by mixing chitosan, agave fructans, gum arabic, and calcium phosphate solutions with vitamin D. The calcium fiber powders were produced in spray-dryer equipment (DL410, Yamato Scientific America Inc., Santa Clara, CA, USA). The spray-drying was performed using an inlet temperature of 180 °C using a dry airflow rate of 0.4 m^3^ min^−1^ at 300 kPa. The outlet temperature was approximately 65 ± 1 °C. *ICa^+^* is a natural calcium-fiber source, including gum arabic (70%), chitosan (1%), and agave fructans (20%), calcium phosphate (4%), and vitamin D (400 UI). A commercial sample of CaCO_3_ was purchased in a local pharmacy (Guadalajara, Mexico). Other reagents were purchased in Sigma Aldrich (Edo. De Mexico, Mexico).

### 2.2. Fourier Transform Infrared Spectroscopy

Fourier transform infrared (FTIR) spectra were measured using a Cary 630 FTIR spectrometer (Agilent Technologies Inc., Santa Clara, CA, USA) equipped with a single-bounce attenuated total reflectance (ATR) diamond crystal interface. Spectra were obtained by triplicate of 48 scans from 4000 to 650 cm^−1^ at a spectral resolution of 4 cm^−1^. The samples were placed on the ATR top plate, pressing the powder sample onto the crystal using a pressure clamp. The background was measured on air to be withdrawn from the raw spectra. Spectral data were observed using Resolutions Pro Software (Varian, Palo Alto, CA, USA).

### 2.3. Apparent Calcium Solubility

The apparent calcium solubility was determined by the ratio of calcium calculated by ionization chromatography and atomic absorption.

Atomic absorption spectroscopy. Approximately 5 g of sample in silica crucibles was subjected to 600 °C in a muffle. After 3 h, the sample was withdrawn and cooled. The residue was treated with 20 mL of 6.5 M HCl and diluted with distilled water to 100 mL. A blank sample and five standard samples (10, 20, 40, 80, and 160 mg/kg) were prepared to quantify the calcium concentration in CaCO_3_ and *ICa^+^*. The samples’ total calcium content was quantified using flame atomic absorption spectroscopy equipment (PerkinElmer, Optima 8300 ICP-OES, Waltham, MA, USA).

Ion Chromatography Analysis. The calcium ionic was quantified, adapting the application note AN-C-040 [29], to determine cations by model 861 Advanced Compact Ion Chromatographer (IC, Metrohm Ltd., Herisau, Switzerland). Briefly, samples were weighed in duplicate (approximately 10 to 11.5 mg) and diluted in 50 mL of deionized and filtered water (0.22 µm). Analysis was performed using the Metrohm suppressor module at a flow of 0.7 mL/min, an average conductivity of 974.4 μS/cm, and an average pressure of 5 MPa. The injection volume was 50 μL at 35 °C. Initially, a calibration curve was developed using a high-purity standard (1000 µg/mL; 99.999%). Cation analysis was performed using a Column Metrosep C6-100/4.0 mm (Metrohm). The mobile phase was a nitric acid/dipicolinic acid solution (1.7:1.7 mM). A standard of calcium phosphate was used to determine ions using 7 standard solutions with different calcium concentrations (1–15 mg/L). The regression coefficient obtained was 0.99.

### 2.4. Cytotoxicity and Mutagenicity Assays

The cytotoxicity assay was carried out in fibroblast cultures cells propagated until reaching 80% of confluency (approximately 10^4^), then 100 mg of *ICa^+^* diluted in PBS (phosphate buffer) were added. The viability was tested after 24 h, and the cells were stained using the trypan blue method. RPMI culture medium was used as a negative control. Cells were observed under the microscope to inspect morphology and discriminate living cells from dead cells [30].

The mutagenicity test was carried out using *Salmonella typhimurium* TA 100 strain [31]. *S. typhimurium* carries a mutation by substituting base pairs in the histidine biosynthesis pathway, dependent on this amino acid. This mutation can be reversed by some agent with which it is possible to detect the mutagenicity of compounds. The lyophilized TA 100 strain was preincubated at 37 °C for 18 h. Subsequently, the samples were inoculated at 1 × 10^9^ UFC/mL (OD_540 nm_ = 0.2) with the test strain. The mixture was poured into Petri dishes in minimal agar medium without histidine and incubated for 48 h. Methyl methanesulfonate mutagen (250 mg/plate) was used as a positive control, and *ICa^+^* was evaluated at 5 mg/plate. The counting of revertant colonies was carried out at the end of the incubation. The test was considered valid when: (1) the number of spontaneous reverting colonies was between 90 and 125 colonies per plate; and (2) the number of reverting colonies in the positive control was more than double compared to the negative control.

### 2.5. Calcium Absorption In-Vivo Experiment

The Animal Committee approved animal care and experimental procedures of CIATEJ (CICUAL (CIATEJ, Mx, ID: 2015-007). Twenty-five 6-week-old female C57/BL6 mice were randomly divided into five groups. Mice were housed in a room with 12-h light/12-h dark periods, relative humidity of 30–60%, and a temperature of 23 ± 1 °C. For one week, animals were fed a standard diet AIN-93M maintenance purified diet (57W5, Pet Foods, Guadalajara, Mexico), and deionized water provided *ad libitum*. The standard diet contained 18.3% protein, 7.1% fat, 5.0% fiber, 63.2% carbohydrates, 0.51% calcium, and 1 UI/g of vitamin D added. After acclimatization, all mice were fed with a calcium-free diet (CFD) for six weeks. The CFD contains the necessary requirements for mice growth, except calcium: 13% protein, 4% fat, 5% fiber, 72% carbohydrates, 0.01% calcium, and 1 UI/g of vitamin D added (CFD, 58M1, Pet Foods). Daily, calcium was supplemented by gavage, using two calcium sources, calcium carbonate and ionic calcium-fiber supplement (*ICa^+^*). Calcium dose was dissolved in 0.1 mL of saline solution and administrated by gavage. Mice were previously deprived of food or water by 4 h. Group 1 (G1) was used as a negative control (CFD without calcium administration), group 2 (G2) was supplemented with 1.3 mg CaCO_3_/day, and group 3 (G3), group 4 (G4), and group 5 (G5) were supplemented with 1.3, 3.3, and 6.6 mg *ICa*^+^/day, respectively. Mice were weighed every week during the experiment. An X-ray study was carried out on mice after six weeks of treatment. Finally, mice were sacrificed for static histomorphometry analyses for bone structure analysis.

### 2.6. X-ray Analysis

The X-ray studies were performed using two concentrations of anesthesia: 1.5% to 2% for maintenance and 4–5% for induction. The anesthetic was isoflurane (a mix of ketamine and phenobarbital). The maintenance was used to keep them relaxed during the X-ray study and finally was applied the induction. The equipment was calibrated per the manufacturer’s instruction protocol. The equipment has a biangular X-ray tube with molybdenum a nose, which applied a potency of 22–40 kVp. Then, mice were set on a tray, ensuring that their column was as straight as possible. The bone of mice was observed using a Hologic DXA scanner (Hologic Corp., Waltham, MA, USA). 200X zooms were made to observe the morphology of bone tissue through X-ray images of mice that remained maintained during the study. The images were viewed, and the length of mice’s tibias and femurs were measured using the CareStream program (Version 10.2 P001, Rochester, NY, USA).

### 2.7. Histomorphometry Analyses (Goldner’s Trichrome Stain)

Left femurs from 25 mice were extracted microsurgically. Then, they were fixed in 10% neutral buffered formalin for 48 h at room temperature. Femurs were transferred to increased ethanol concentrations from 70% to absolute. Afterward, the bones were cleared with xylene, and the bone specimens were embedded in methyl methacrylate acrylic. Five micron-thickness bone sections were cut using a microtome until mid-depth section with a slightly curved growth plate, roughly around ~500 microns to reach that depth. The bones were not decalcified, and specimens were used for Mason Goldner’s trichome stain. Bone histomorphometry was made from the complete histological image (×50). Samples were analyzed with a QImaging camera, a 12-bit Color, Retiga Camera (32-00013B-157, Canada). The region of interest (ROI) for bone histomorphometry was set at1 mm off the peak and 250-micron border on the growth plate. The scans (*n* = 25) depict the structural changes, which were described by the nomenclature, abbreviations, and parameters following the recommendations of the American Society for Bone and Mineral Research (ASBMR) [32].

### 2.8. Statistical Analysis

Data were expressed as mean ± SEM. The student’s T-test was performed, differences were considered significant if *p* < 0.05. Statistical analyses were performed using Statgraphics Centurion XV statistical software (v. 2.15.06, Statpoint Technologies, Inc., Warrenton, VA, USA). The reported analysis was the average of three independent measurements. For the statistical analysis of ration bone (BV/TV), trabecular thickness (Tb. Th), trabecular space (Tb. Sp), and trabecular number (Tb N), one-way ANOVA were performed for multiple comparisons, Tukey’s test was applied, values were considered significant when *p* < 0.05, since higher (****) to lower significance (*).

## 3. Results and Discussion

### 3.1. Fourier Transform Infrared Spectroscopy

**Figure 1:**
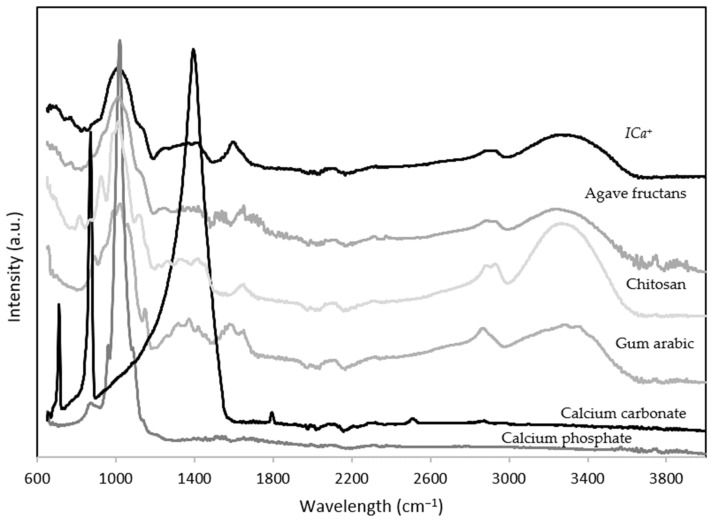
Fourier-transform infrared spectroscopy (FTIR) spectrum of calcium carbonate, calcium phosphate, chitosan, gum arabic, agave fructans, and ionic calcium-fiber supplement (*ICa*^+^).

The FTIR spectra of the calcium carbonate, calcium phosphate, agave fructans, gum arabic, chitosan, and *ICa*^+^ are shown in Figure 1. Calcium carbonate displayed three strong asymmetric bands at 713, 872, and 1400 cm^−1^. Also, calcium carbonate displayed a small symmetrical vibration band at 1798 cm^−1^ associated with the crystalline state. Similar bands were reported by Rodriguez–Blanco et al. [33] for calcium carbonate samples.

Calcium phosphate showed an intense band at 1023 cm^−1^ and two weak bands at 1079 and 972 cm^−1^ related to the asymmetric vibrations of the phosphate groups. Chitosan FTIR spectra showed broadband from 3650–3000 cm^−1^ associated with vibrations of the hydroxyl and amine groups. Bands at 1656 and 1595 cm^−1^ were correlated with amide I and amide III stretching vibrations. Bands at 1150, 1030, and 998 cm^−1^ correspond to the symmetric stretching of glycosidic bonds (C–O and C–O–C) [34]. Gum arabic showed broadband at 3330 cm^−1^ of the hydroxyl groups. Asymmetric and symmetric stretching vibrations of the glucuronic acids were observed at 1602 and 1430 cm^−1^ and stretching of C–O bond bands at 1280 and 1010 cm^−1^. Also, a band at 823 cm^−1^ was associated with the pyranose group [35]. Agave fructans presented broadband from 3650–3000 cm^−1^ associated with vibrations of the hydroxyl groups. Stretching asymmetric vibrations were due to C–H bonds. Agave fructans fingerprint was observed in the region of 1192–900 cm^−1^ represented for C–C, C–O stretching and C–O–H, C–O–C bending. Velazquez–Martínez et al. [36] reported similar bands in polysaccharides from *Agave angustifolia* Haw.

The FT-IR spectrum of the *ICa^+^* showed typical bands of complex carbohydrates that composite it. The *ICa^+^* contains calcium phosphate and polysaccharides, including gum arabic, agave fructans, and chitosan. An absorption band from 3650–3000 cm^−1^ was associated with the stretching vibration of OH and NH groups. Bands observed at 2500 to 3000 cm^−1^ corresponded to stretching vibration of the C–H bond. An absorption band attributed to C=O vibration of the acetylated units of chitosan at 1635 cm^−1^. The lack of intense absorption bands of the *ICa^+^* in the region from 2000–1100 cm^−1^ probably resulted from the electrostatic interactions between functional groups of the ingredient. The FTIR spectrum showed strong bands in the region between 1200 cm^−1^ and 800 cm^−1^ related to polysaccharides. A typical band of phosphate group appears faintly on the spectrogram of the calcium-stabilizing *ICa*^+^ complex, suggesting that salt can be found in its amorphous form. In postmenopausal women, a calcium supplementation study showed that amorphous calcium carbonate had better absorption than crystalline calcium [37].

### 3.2. Apparent Calcium Solubility in the Supplements

Apparent calcium solubility was quantified using the ratio between the calcium concentration estimated by ion chromatography (IC) and the calcium concentration estimated by atomic absorption spectroscopy (AAS). IC is a technique used to determine inorganic and organic ions, including Na^+^, K^+^, Cl^−^ NO_2_^−^, NH_4_^+^, Ca^2+^, Mg^2+^, or Cr^3+^, Cr^6+^, etc. [38]. The results showed that the CaCO_3_ sample had approximately 39,266 ± 345 mg Ca^2+^/kg. On the other hand, the AAS technique was used efficiently to quantify the concentration of elements in foods and beverages from part per million (mg/L) to part per billion (µm/L) [39]. As expected, calcium carbonate had approximately 318,400 ± 320 mg Ca^2+^/kg (31.8%). Approximately the average mass of a CaCO_3_ tablet has 1850 mg, of which 600 mg is elemental calcium. A pure sample of CaCO_3_ contains approximately 40% calcium. However, commercial tablets contain adjuvants for tablet formation that relatively decrease the percentage of calcium. On the other hand, the *ICa^+^* sample showed a calcium concentration of approximately 16,450 ± 500 mg/kg. AAS results confirmed that the CaCO_3_ sample had 19.3-fold more elemental calcium than that of the *ICa^+^* sample.

Ionic calcium concentration from calcium carbonate calculated by IC was eight folds less than that calcium estimated by AAS. Thus, an apparent solubility reduction of the calcium carbonate was approximately 87.7%. Calcium carbonate has extremely low solubility in water, so this behavior was related to the amount of calcium carbonate sample that passed through the 0.22 μm membrane during the filtration process. However, the calcium concentration is relatively low compared to the initial concentration in the sample.

On the other hand, the amount of Ca^2+^ contained in the *ICa^+^* sample was 16,510 ± 720 mg Ca^2+^/kg. This result showed that calcium ions were soluble in the sample, indicating an apparent solubility of approximately 100%. The *ICa*^+^ complex behaves like a polyelectrolyte macromolecule that, in an aqueous (weak acids and neutral) solution, equilibrates with the environment surrounding it in such a way that in the solution that it gives up calcium ions available in the medium. This characteristic is vital for maintaining the solubility of calcium ions in intestinal conditions. As mentioned, Ca^2+^ is absorbed by specialized cells in the small intestine by transcellular transport and paracellular transport. *ICa^+^* sample appears to behave like a polyelectrolyte that dissolves in an aqueous medium, producing polyions and counter ions that favor the dissolution of calcium [40,41]. One of the significant factors complicating calcium salts’ bioavailability is the influence of pH on the dissolution process [42].

### 3.3. Cytotoxicity and Mutagenicity In Vitro

*ICa^+^* safety was evaluated by in vitro cytotoxicity (fibroblast) and mutagenicity assays (bacterial reverse test). In vitro trypan blue exclusion evaluated the viable cells in close contact with the *ICa^+^* (biocompatibility). The results showed 100% cell viability, indicating that the *ICa^+^* did not display a cytotoxic effect on cell lines. If the *ICa*^+^ sample contained a compound that can alter or disrupt cell membrane activity, it could lead to cell death (blue staining) [43,44]. For example, Rodrigues et al. [43] reported that some chitosan materials showed a low cytotoxic effect because the cationic chitosan groups can interact with cell membranes altering some metabolic functions.

The genotoxicity of a substance is measured by changes in cellular behavior and the damage caused to DNA [45]. *ICa^+^* mutagenic effect was evaluated by the Ames test of the *S. typhimurium*. The number of revertant colonies observed on the *ICa^+^* supplement was approximately 95.7 + 7.8, lower than those expressed by the negative control 152.3 + 8.9. In comparison, the ethyl methanesulfonate expressed several reverting colonies, approximately 556.1 + 13.1 (positive control). Our results suggested that the *ICa^+^* supplement had no mutagenic effects in the *S. typhimurium* TA100 strain.

### 3.4. Calcium Absorption In Vivo Mice Model

**Figure 2:**
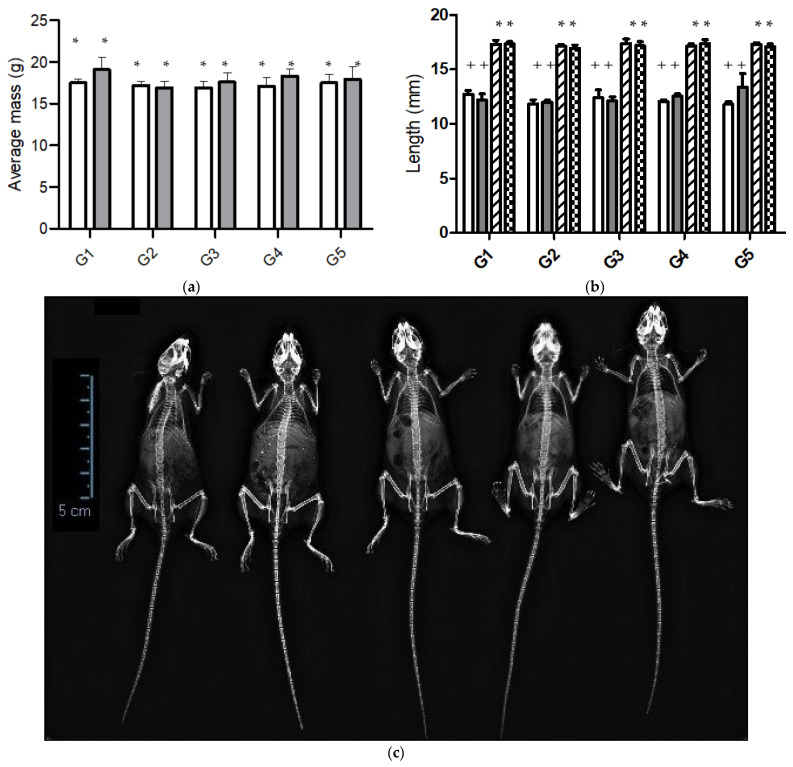
(**a**) Body mass of C57/BL6 mice fed diets differing in calcium content for 6 weeks: G1 (CFD), G2 (CFD + CaCO_3_), G3 to G5 (CFD + *ICa^+^*); (**b**) femur and tibia lengths of mice groups (G1–G5) estimated by X-ray images at end of the experiment. Data correspond to left tibia (white bar), right tibia (gray bar), left femur (forward bar), and right femur (square bar); (**c**) X-ray images of mice from groups G1 to G5 (from left to right). Significant difference in each column is expressed as different symbols (* & +; *p* < 0.05).

Calcium is an essential nutrient for the development and growth of living things. A calcium-free diet (CFD) produces calcium resorption from bones to maintain the plasma calcium concentration, generating a natural decalcification model [6]. In the present study, we compared changes in bone mass and microarchitecture in mice groups fed during six weeks with CFD (without calcium supplementation), CFD plus CaCO_3_, and CFD plus three different *ICa^+^*. Average masses of mice fed with different diets at the beginning and the end of the experiment are shown in Figure 2a. At the beginning of the experiment, all groups had an average mass of 17.2 ± 0.3 g without showing a significant statistical difference between experimental groups. After the six weeks, the difference in initial and final average mass does not go beyond 2.5 g, which means an increase of 14.3%.

Mice mass showed no statistically significant difference (*p* < 0.05) in the different groups at the end of the experiment. However, the average mass of mice fed a CFD plus CaCO_3_ was 5% lower than those fed a CFD plus *ICa^+^* and 10% lower than the CFD. Similar results were observed by Somerville et al. [46], who reported that the growth of female C57/BL6 mice of 13 weeks old fed with standard diet was about 20 ± 1 g. Also, Zhang et al. [47] reported that mice fed with different amounts of calcium did not show a difference in body mass.

We used the X-ray imaging technique to show the complete structure for the entire mice bones in all study groups (Figure 2c). The results showed that mice absorbed calcium in the food supplements. In general, mice were not different at this time of experimentation. It displays most of the bones of mice, highlighting the pelvis, femurs, tibia, spine, and skull. Mice’s femurs and tibias were measured to compare bone growth (Figure 2b). The results showed no significant difference in the femurs and tibias of the mice groups. That is, the length of the tibia and femur was developed. The main consequence of a diet low or limited in calcium is the inherent loss of bone mass density. The microstructure of porous bone is the primary reserve for the beginning of calcium reabsorption for the body to preserve its vital functions. According to McNamara [48], the trabecular bone is a highly porous structure of bone tissue interconnected by rods and plates of spongy appearance.

**Figure 3:**
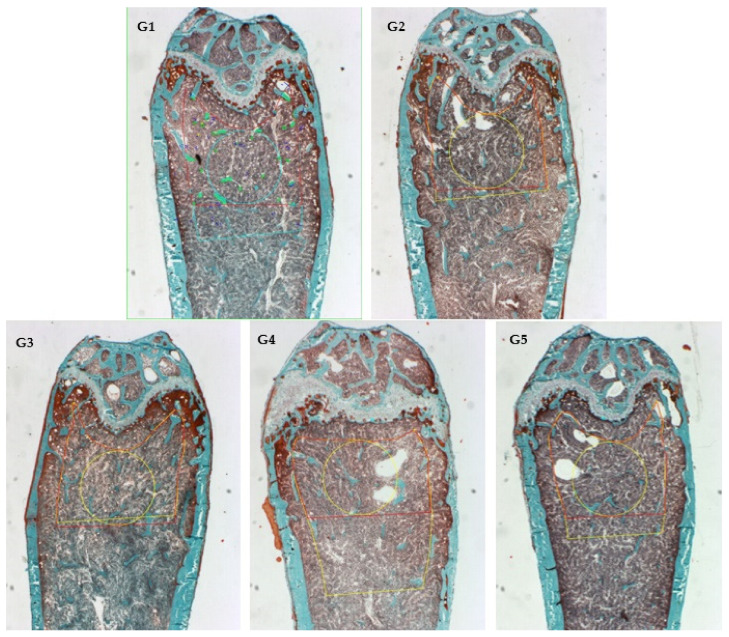
Sagittal Goldner’s stain images of distal femur were used in histomorphometric analysis: G1 (mice fed CFD), G2 (CFD + CaCO_3_), G3 to G5 (CFD + *ICa^+^*).

**Figure 4:**
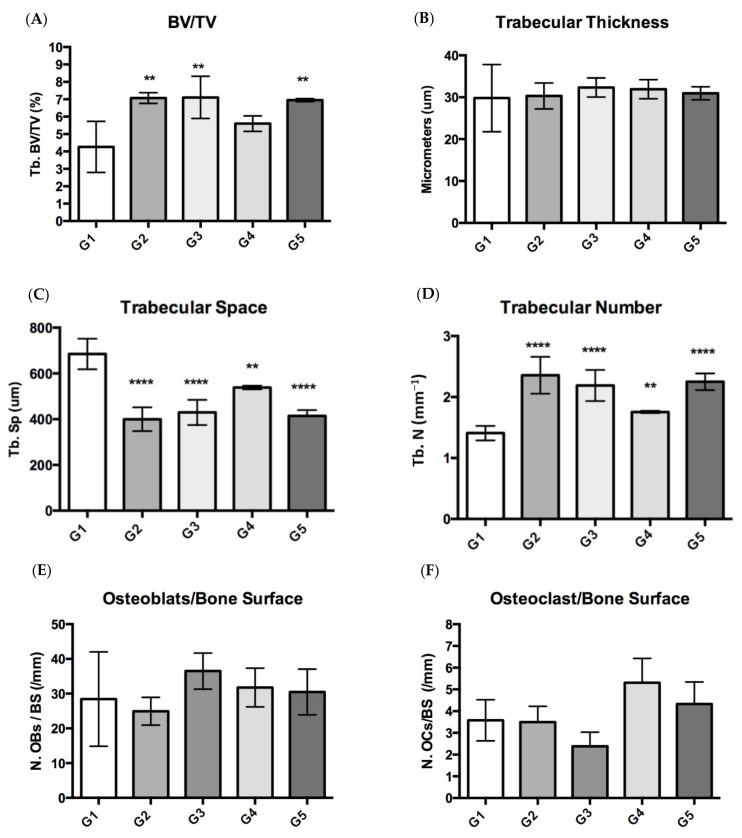
Histomorphometric bone parameters from mice groups: G1 (mice fed CFD), G2 (CFD + CaCO_3_), and G3 to G5 (CFD + *ICa^+^*). (**A**) Bone volume fraction; (**B**) trabecular thickness (Tb.Th); (**C**) trabecular space (Tb.Sp); (**D**) trabecular number (Tb.N); (**E**) osteoblast/bone surface; and (**F**) osteoclast/bone surface. Values were considered significant when *p* < 0.05, since higher (****) to lower significance (*).

Masson–Goldner trichrome staining was used for the histomorphometry evaluation (Figure 3). Images from sagittal sections of the femurs (distal metaphysis) dyed green showed the percentage of newly formed mineralized bone volume contrasted with total tissue volume (BV/TV) in the experimental groups supplemented with CFD plus *ICa^+^*, G3 to G5. Figure 4 shows the histomorphometric evaluation results by quantifying bone material in a given area (ROI). The bone material was heterogeneously distributed, particularly in the cancellous bone area for G2–G5. Figure 4A showed that bone volume (BV/TV) was similar and comparable for G2 (CFD + CaCO_3_) and G3–G5 (CFD + *ICa^+^*), but higher than G1 (CFD) for all supplemented groups. The BV/TV is an essential microstructural parameter of the new generation of bone, which was documented in papers [49,50,51]. This is a relevant result because *ICa^+^* can promote de novo bone production in the mice model implemented, i.e., calcium supplementation with CaCO_3_ or *ICa^+^* for 6 weeks preserved a similar microstructure of the mice’s bone tissue. There are experiments with similar time of treatment, such as six weeks, as shown by Berman et al. [49] with Raloxifene (a selective estrogen receptor modulator), which demonstrated efficacy in preventing osteoclastic activity and promoting osteoblastic activity.

Increases in cancellous bone volume fraction were heavily driven by increases in trabecular number, which is relevant because bone tissues trabecular bone has a high turnover compared with the cortical bone. The trabecular numbers (Tb. N) in the experimental groups were higher than in the G1 group (CFD). It was impossible to elucidate a difference concerning the thickness of the trabecular bone due to the 2D nature of the images analyzed in the histomorphometry assay (Masson-Goldner), so no significant differences were observed. Nevertheless, the trabecular space (Tb. Sp) in the G1 group was much more extensive compared with the area evaluated (mm^−1^), which makes sense because it showed a more significant space and a relatively lower trabecular number (Tb. N) in a specific area (ROI). Although, osteoclast and osteoblast numbers did not show a statistical difference between groups by this approach using a static histomorphometric assay. These results cannot be conclusive for their technical limitation. There is a better way to quantify them by TRAP stained and analysis using higher magnification; as well, the osteoblast activity could be analyzed by marker genes, such as Ibsp (encoding bone sialoprotein), Alpl (encoding tissue nonspecific alkaline phosphatase), and Bglap (encoding osteocalcin).

The model employed describes the behavior of young adult mice that present severe malnutrition in calcium intake. Some research like Ferretti et al. [52] reported that 3-month-old Sprague–Dawley male rats fed for four weeks with a calcium-deprived diet developed osteoporosis in vertebras and femurs due to enhanced bone resorption. Although the results presented in this work were not performed by microcT, the most used tool for this type of measurement, it was performed by histomorphometry in bone tissue samples and performing quantifications in a selected area (ROI), some articles show that the results by microcT and with histomorphometric analysis in samples stained are equally valuable [53].

## 4. Conclusions

The study focused on an ionic calcium-fiber supplement’s physicochemical and biological evaluation. The infrared spectroscopy results show a mainly characteristic structure of the polysaccharides that make up the sample. The apparent solubility test showed that the polysaccharides contend in the ionic calcium-fiber supplement increases the solubility of the calcium ion. The ionic calcium-fiber supplement did not present cytotoxic and genotoxic activities in vitro.

The model of bone decalcification of young mice caused by a calcium-free diet was successfully established after six weeks of supplementations. The animal showed a ratio of BV/TV similar in calcium carbonate mice and ionic calcium-fiber supplement and was lower in the calcium-deprived control group. However, experiments were only carried out across six weeks. These results show that ionized calcium supplements can be safely employed to promote the absorption and retention of osseous calcium.

## Data Availability

The data presented in this study are available on request from the corresponding author.
